# Supplementary material for: Breaking the norm: population-scale deviations of brain structure in depression and anxiety
Source: Mol Psychiatry. 2026 Jul 18;31(10):6034–45. doi: 10.1038/s41380-026-03691-4 (PMC13569451; doi:10.1038/s41380-026-03691-4)
Supplement: Supplementary file 1 [file 41380_2026_3691_MOESM1_ESM.pdf]

Breaking the Norm: Population-Scale Deviations of Brain Structure in Depression and Anxiety
Supplementary Material

Julius Wiegert[1,2], Sebastián Marty-Lombardi[1,2], Jailan Oweda[1,2], Esra Lenz[1,2], Peter Ahnert[3], Klaus Berger[4], Hermann Brenner[5, 6], Josef Frank[7], Hans J. Grabe[8], Karin Halina Greiser[9], Johanna Klinger-König[8], André Karch[4], Michael Leitzmann[10], Claudia Meinke-Franze[11], Rafael Mikolajczyk[12,13], Frauke Nees[14,15,16], Thoralf Niendorf[17], Oliver Sander[18], Carsten Oliver Schmidt[11], Steffi G. Riedel-Heller[19], Kerstin Ritter[20,21,22], Annette Peters[23,24,25], Tobias Pischon[26,27,28], Stephanie Witt[7,29], Johannes Nitsche[1,2], Joonas Naamanka[1,2,30], Sebastian Volkmer[1,2], Antonia Mai[1,2], Amrou Abas[1,2], Xiuzhi Li[1,2], Andreas Meyer-Lindenberg[2], Tobias Gradinger[1,2], Fabian Streit[1,2,29], Urs Braun[1,2], and Emanuel Schwarz*[1,2,29]

[1]Hector Institute for Artificial Intelligence in Psychiatry, Central Institute of Mental Health, Medical Faculty Mannheim, Heidelberg University, Mannheim, Germany
[2]Department of Psychiatry and Psychotherapy, Central Institute of Mental Health, Medical Faculty Mannheim, Heidelberg University, Mannheim, Germany
[3]Universität Leipzig, Institute for Medical Informatics, Statistics and Epidemiology
[4]Institute of Epidemiology and Social Medicine, University of Münster, Münster, Germany
[5]German Cancer Research Center (DKFZ), Heidelberg, Germany
[6]Network Aging Research, Heidelberg University, Heidelberg, Germany
[7]Department of Genetic Epidemiology in Psychiatry, Central Institute of Mental Health, Medical Faculty Mannheim, Heidelberg University, Mannheim, Germany
[8]Department of Psychiatry and Psychotherapy, University Medicine Greifswald, Greifswald, Germany
[9]Division of Cancer Epidemiology, German Cancer Research Center (DKFZ), Heidelberg, Germany
[10]Department of Epidemiology and Preventive Medicine, University of Regensburg, Regensburg, Germany
[11]Institute for Community Medicine, University Medicine Greifswald, Greifswald, Germany
[12]Institute for Medical Epidemiology, Biometrics, and Informatics, Medical Faculty of the Martin Luther University Halle-Wittenberg, Halle (Saale)

[13]German Center for Mental Health (DZPG), partner site Halle-Jena-Magdeburg, Halle (Saale), Germany
[14]Institute of Medical Psychology and Medical Sociology, University Medical Center Schleswig-Holstein, Kiel University, Kiel, Germany
[15]Department of Child and Adolescent Psychiatry and Psychotherapy, Central Institute of Mental Health, Medical Faculty Mannheim, Heidelberg University, Mannheim, Germany
[16]Institute of Medical Psychology, Faculty of Medicine, Ludwig-Maximilians-Universität München, Munich, Germany
[17]Berlin Ultrahigh Field Facility (B.U.F.F.), Max Delbrueck Center for Molecular Medicine in the Helmholtz Association (MDC), Berlin, Germany
[18]Clinic for Rheumatology and Hiller Research Center, University Hospital, Heinrich-Heine-University Düsseldorf, Germany
[19]Institute of Social Medicine, Occupational Health and Public Health (ISAP), Leipzig University, Leipzig, Germany
[20]Department of Machine Learning, Hertie Institute for AI in Brain Health, University of Tübingen, Germany
[21]Department of Psychiatry and Neurosciences, Charité – Universitätsmedizin Berlin (corporate member of Freie Universität Berlin, Humboldt-Universität zu Berlin, and Berlin Institute of Health), Berlin, Germany
[22]Tübingen AI Center, Tübingen, Germany
[23]Institute of Epidemiology, Helmholtz Zentrum München - German Research Center for Environmental Health (GmbH), Neuherberg, Germany
[24]Chair of Epidemiology, Institute for Medical Information Processing, Biometry and Epidemiology, Medical Faculty, Ludwig-Maximilians-Universität München, Munich, Germany
[25]German Center for Mental Health (DZPG), partner site Munich, Munich, Germany
[26]Max Delbrück Center for Molecular Medicine in the Helmholtz Association (MDC), Molecular Epidemiology Research Group, Berlin, Germany
[27]Charité - Universitätsmedizin Berlin, corporate member of Freie Universität Berlin and Humboldt-Universität zu Berlin, Berlin, Germany
[28]Max Delbrück Center for Molecular Medicine in the Helmholtz Association (MDC), Biobank Technology Platform, Berlin, Germany
[29]German Center for Mental Health (DZPG), Partner Site Mannheim - Heidelberg - Ulm, Germany
[30]SleepWell Research Program, Faculty of Medicine, University of Helsinki, Helsinki, Finland

May 2026

*Corresponding author

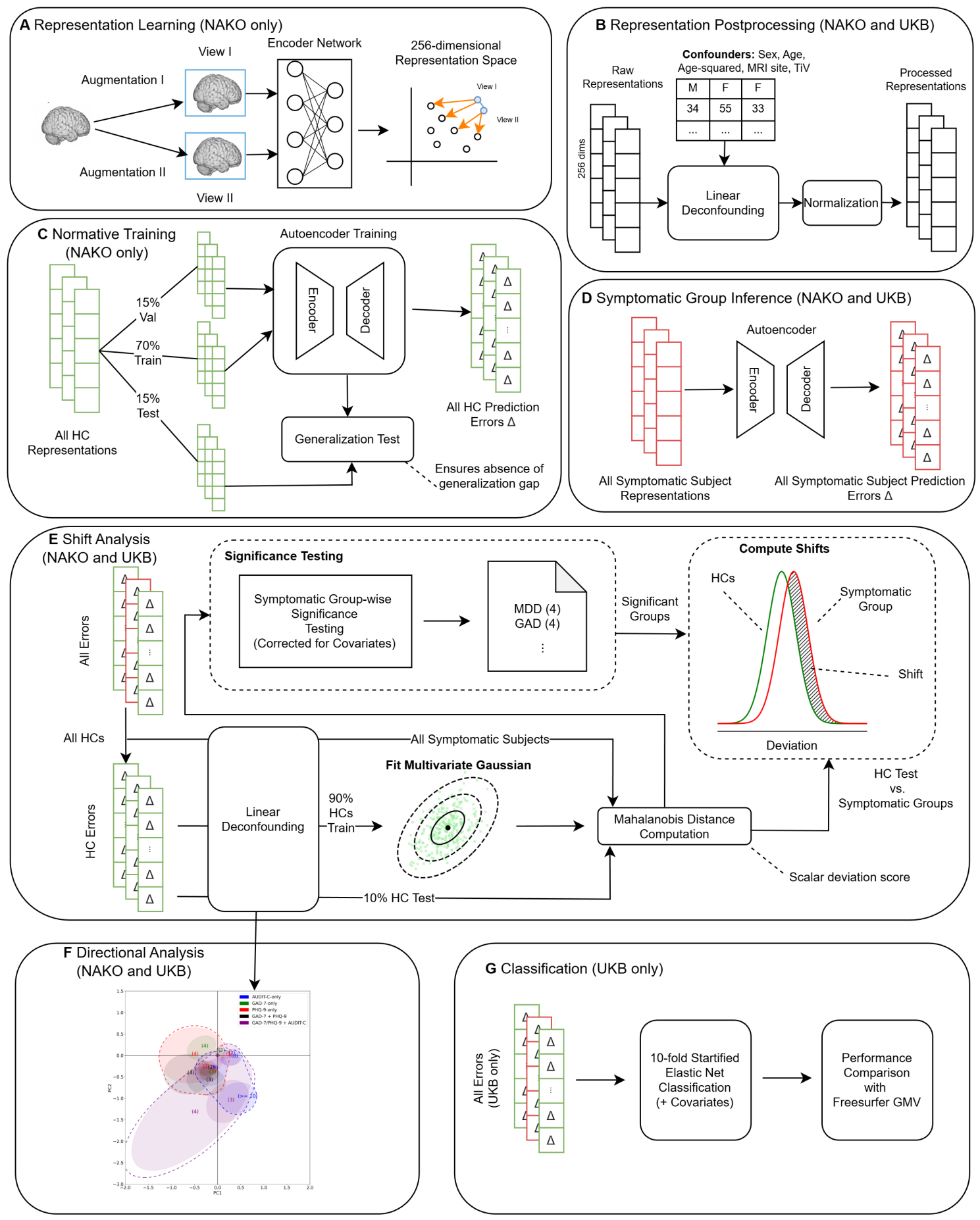

Figure 1: Complete study workflow. (**A**) Representation learning: brain MRI data were augmented and encoded into a 256-dimensional representation space using a contrastive learning framework. (**B**) Representation postprocessing: embeddings were linearly deconfounded for sex, age, age-squared, MRI site, and total intracranial volume (TIV), followed by normalization. (**C**) Normative training: an autoencoder was trained on healthy controls (HCs) with a train–validation–test split to predict HC representations, ensuring no generalization gap. (**D**) Symptomatic inference: deviations (Δ) were obtained for all symptomatic subjects by applying the HC-trained autoencoder. (**E**) Shift analysis: group-level deviations were compared against HC variability using multivariate Gaussian modeling, Mahalanobis distance, and bootstrap significance testing corrected for covariates. (**F**) Directional analysis: deviations were projected via principal component analysis (PCA). (**G**) Classification analysis in the UKB.

1 Data Filtering

1.1 NAKO

Field Name	Description	Missing Value Indicators	Affected
a_emo_miniscr_dp	MINI Screening MDD	7777	226
a_emo_phq9_kat	PHQ-9 category	7777	1 240
a_emo_gad7_dia	GAD-7 category	7777	1 285
a_alk_audit_c	AUDIT-C sum score	-99	1 285

Table 1: Missing values in key phenotypic fields from the NAKO cohort (German National Cohort). Field names correspond to raw database variables: `a_emo_miniscr_dp`, MINI International Neuropsychiatric Interview (MINI) screening for major depressive disorder (MDD); `a_emo_phq9_kat`, Patient Health Questionnaire-9 (PHQ-9) categorical score; `a_emo_gad7_dia`, Generalized Anxiety Disorder-7 (GAD-7) categorical score; `a_alk_audit_c`, Alcohol Use Disorders Identification Test–Consumption (AUDIT-C) sum score. "Missing value indicators" list the coding used for missing data in the raw dataset, and "Affected" gives the number of participants with missing entries.

1.2 UKB

1.2.1 Differing Standard Unit Definitions

In UK Biobank, alcohol units were defined as follows: pint or can of beer/lager/cider = 2 units; single shot of spirits (25 ml) = 1 unit; small glass of fortified wine = 1 unit; standard glass of wine (175 ml) = 2 units; large glass of wine (250 ml) = 3 units; bottle of wine (75 cl) = 9 units.

In NAKO, a standard drink corresponded to one small bottle or glass of beer (0.33 l), one small glass of wine or sparkling wine (0.125 l), or one simple shot of spirits (2 cl).

In general, the alcohol concentration per unit was lower in UKB compared to NAKO, which likely accounts for the higher proportion of participants with AUDIT-C ≥ 10 in UKB relative to NAKO (NAKO: 64% AUDIT 8, 25% AUDIT 9, 11% AUDIT ≥ 10; UKB: 40%, 29%, 30%).

1.2.2 Neurodegenerative Exlcusion

ICD-10 Code	Condition
G30	Alzheimer's Disease
G20	Parkinson's Disease
G10	Huntington's Disease
G12.2	Amyotrophic Lateral Sclerosis (ALS)
G35	Multiple Sclerosis
I60–I64	Stroke
G45.9	Transient Ischemic Attack (TIA)
S06	Traumatic Brain Injury (TBI)
M79.7	Fibromyalgia
G25.0	Essential Tremor
G24	Dystonia
G61.0	Guillain-Barré Syndrome
G36.0	Neuromyelitis Optica
B20	HIV-associated neurocognitive disorders
C71	Brain tumor
G91	Hydrocephalus

Table 2: ICD-10 codes used to exclude participants with neurodegenerative, neurological, or related conditions from the UKB (UK Biobank) instance two MRI cohort. Conditions include major neurodegenerative diseases (e.g., Alzheimer's disease, Parkinson's disease, Huntington's disease, amyotrophic lateral sclerosis [ALS], multiple sclerosis), cerebrovascular events (stroke, transient ischemic attack [TIA]), traumatic brain injury (TBI), and other neurological disorders listed.

1.2.3 Filtering Summary

Step	Filtering Step	Remaining	Removed	Reason
1	Initial Sample	43 248	–	–
2	FreeSurfer QC	39 369	3 879	Any missing FreeSurfer statistics
3	GAD-7 Filter	29 854	9 515	Any missing GAD-7 question
4	PHQ-9 Filter	25 977	3 877	Any missing PHQ-9 question
5	AUDIT-C Filter	25 977	0	Any missing AUDIT-C question

Table 3: Sequential filtering of the UKB (UK Biobank) MRI instance two cohort. "Remaining" indicates the number of participants retained after each step, and "Removed" gives the number excluded at that stage. FreeSurfer QC = exclusion of participants with missing FreeSurfer-derived brain structural measures; GAD-7 (Generalized Anxiety Disorder-7), PHQ-9 (Patient Health Questionnaire-9), and AUDIT-C (Alcohol Use Disorders Identification Test–Consumption) filters = exclusion of participants with missing responses to any item of the respective questionnaires.

1.2.4 UKB Questionnaire Fields

Field ID Showcase	Description	Missing Value Indicators
p120112	PHQ-9 item	"Prefer not to answer" or *null*
p120111	PHQ-9 item	"Prefer not to answer" or *null*
p120110	PHQ-9 item	"Prefer not to answer" or *null*
p120109	PHQ-9 item	"Prefer not to answer" or *null*
p120108	PHQ-9 item	"Prefer not to answer" or *null*
p120107	PHQ-9 item	"Prefer not to answer" or *null*
p120106	PHQ-9 item	"Prefer not to answer" or *null*
p120105	PHQ-9 item	"Prefer not to answer" or *null*
p120104	PHQ-9 item	"Prefer not to answer" or *null*

Table 4: UK Biobank field IDs corresponding to items of the PHQ-9 (Patient Health Questionnaire-9) depression scale. Each field represents a single questionnaire item. Missing values are coded as either "Prefer not to answer" or left as null entries in the dataset.

**Field ID Showcase**	**Description**	**Missing Value Indicators**
p29058	GAD-7 item	"Prefer not to answer" or *null*
p29059	GAD-7 item	"Prefer not to answer" or *null*
p29060	GAD-7 item	"Prefer not to answer" or *null*
p29061	GAD-7 item	"Prefer not to answer" or *null*
p29062	GAD-7 item	"Prefer not to answer" or *null*
p29063	GAD-7 item	"Prefer not to answer" or *null*
p29064	GAD-7 item	"Prefer not to answer" or *null*

Table 5: UK Biobank field IDs corresponding to items of the GAD-7 (Generalized Anxiety Disorder-7) scale. Each field represents a single questionnaire item. Missing values are coded as either "Prefer not to answer" or left as null entries in the dataset.

**Field ID**	**Description**	**Missing Value Indicators**
p20414	Alcohol consumption frequency	"Prefer not to answer" or *null*
p20403	Number of drinks consumed on a drinking day	"Prefer not to answer" or *null*
p29093	Frequency of consuming six or more units of alcohol	"Prefer not to answer" or *null*

Table 6: UK Biobank field IDs corresponding to items of the AUDIT-C (Alcohol Use Disorders Identification Test–Consumption). Each field represents one questionnaire item. Missing values are coded as either "Prefer not to answer" or left as null entries.

2 Technical Details

2.1 Contrastive Feature Extraction

Normative modeling (NM) requires embedding brain magnetic resonance imaging (MRI) images into a suitable representational space that captures meaningful variations in brain structure. To achieve this, we use momentum contrast (MoCo) [1] for self-supervised feature extraction, reducing the dimensionality of grey matter volume (GMV) images to 256 dimensions.

MoCo implements contrastive learning by encouraging similar representations for augmented versions of the same image (positive pairs) while pushing apart representations of other images (negative pairs). It maintains a dynamic dictionary of encoded images (size 8 192) via a momentum encoder, ensuring a rich and diverse set of negative pairs, which is crucial for effective contrastive learning. By decoupling the number of negative pairs from batch size, MoCo remains scalable and efficient.

We evaluated three candidate embedding dimensionalities (128, 256, 512) during MoCo pre-training. Although a systematic optimization was not feasible due to the high computational cost of retraining MoCo with different settings—particularly because the augmentation pipeline is CPU-bound, cannot be precomputed, and requires on-the-fly random sampling—256 dimensions consistently yielded the smoothest and most stable loss convergence. In contrast, 128 and 512 dimensions showed slower or less stable convergence under otherwise identical settings. We further note that the optimal embedding size is not independent of other hyperparameters. In particular, the contrastive learning temperature and several architectural choices can interact with dimensionality to influence convergence and representation quality. Consequently, 256 dimensions was selected as a pragmatic choice balancing computational constraints and convergence stability. Furthermore, this dimensionality is also commonly used in prior contrastive learning work and balances representational capacity with downstream model complexity [2, 3, 4].

Unlike autoencoders (AEs), which prioritize input reconstruction, MoCo directly optimizes embedding similarity and dissimilarity, producing more structured representations. Additionally, it avoids the need for bottleneck tuning or reconstruction loss adjustments, making it a robust choice for feature extraction. Furthermore, in our experiments, AEs were unable to embed the data into meaningfully compact representations as effectively as MoCo. When constrained to smaller latent spaces, they either suffered from mode collapse or exhibited poor reconstruction quality.

To generate positive pairs, we apply two different random augmentations to the same original image, ensuring that both views retain the core anatomical features while introducing small variations. These augmentations—random affine transformations, random gamma adjustments, Gaussian noise, Gaussian blur, and random elastic deformations—help the model learn invariance to minor differences while still recognizing that both augmented views originate from the same underlying brain scan. We use the TorchIO library [5] to apply these transformations to the fully processed images.

Our MoCo encoder is a lightweight convolutional network implemented in PyTorch [6] consisting of five convolutional blocks, each followed by ReLU activation and max pooling (stride = 2) for downsampling, and three fully connected layers for gradual downsampling. The full architecture is detailed in Table 7.

Block	Layer Type	Kernel Size	Channels	Stride	Activation
Block 1	Conv3D	$3 \times 3 \times 3$	$1 \rightarrow 32$	1	ReLU
	MaxPool3D	$3 \times 3 \times 3$	-	2	-
Block 2	Conv3D	$3 \times 3 \times 3$	$32 \rightarrow 32$	1	ReLU
	MaxPool3D	$3 \times 3 \times 3$	-	2	-
Block 3	Conv3D	$3 \times 3 \times 3$	$32 \rightarrow 64$	1	ReLU
	MaxPool3D	$3 \times 3 \times 3$	-	2	-
Block 4	Conv3D	$3 \times 3 \times 3$	$64 \rightarrow 64$	1	ReLU
	MaxPool3D	$3 \times 3 \times 3$	-	2	-
Block 5	Conv3D	$3 \times 3 \times 3$	$64 \rightarrow 64$	1	ReLU
Fully Connected	Linear	-	$8000 \rightarrow 512$	-	ReLU
	Linear	-	$512 \rightarrow 256$	-	ReLU
	Linear	-	$256 \rightarrow 256$	-	-

Table 7: Architectural overview of the MoCo (Momentum Contrast) encoder convolutional neural network used for representation learning on 3D brain MRI data. Conv3D = three-dimensional convolutional layer; MaxPool3D = three-dimensional max-pooling layer; Linear = fully connected layer; ReLU = rectified linear unit activation function. Channels indicate input and output feature maps for each layer.

2.2 Deconfounding Strategy

Many of the differences captured by MoCo appear to be influenced by confounding factors (Figure 2), which may obscure the underlying pathological signal. Therefore, we apply linear deconfounding using linear regression residualization with sex (one-hot encoded), age, age-squared, total intracranial volume (TiV), and recruitment center (one-hot encoded) as confounders. TiV was extracted using the TiV estimated by FreeSurfer [7]. We perform this linear deconfounding on each of the 256 MoCo dimensions. As shown in Figure 2, the magnitude of significant Spearman correlations between embeddings and confounders is markedly reduced after deconfounding, indicating effective removal of confounding effects.

We also explored more complex deconfounding strategies, such as conditional AEs and adversarial deconfounding, but found the optimization procedure to be diverging, likely due to the strong and pervasive nature of confounding in the dataset. As a result, we opted for rigorous linear deconfounding, potentially at the cost of some signal loss, with the aim of reducing—though not entirely eliminating—confounding bias in downstream analyses.

2.3 Normative Modeling

We use the deconfounded MoCo embeddings as input for our NM pipeline, performing all modeling exclusively on the HC data. First, we min-max scale the embeddings to ensure stability during subsequent modeling. We then employ a simple 8-hidden-layer AE with ReLU activations to model normality of the deconfounded, scaled MoCo embeddings. Table 8 describes the AE architecture in detail.

The AE is trained to minimize the mean squared error (MSE) reconstruction loss. We use the AdamW optimizer (learning rate 0.0005) with batch size 64 for 300 epochs with early stopping based on validation loss (patience = 20), compressing the data into a latent representation of size

Layer	Type	Output Dimension
Input	-	256
Block 1	Linear	150
	ReLU	150
Block 2	Linear	100
	ReLU	100
Block 3	Linear	75
	ReLU	75
Block 4	Linear	50
	ReLU	50
Block 5	Linear	75
	ReLU	75
Block 6	Linear	100
	ReLU	100
Block 7	Linear	150
	ReLU	150
Block 8	Linear	256
	Sigmoid	256

Table 8: Architecture of the normative autoencoder used for reconstruction of deconfounded brain MRI embeddings. The network consists of eight fully connected (linear) blocks with symmetric compression and expansion, mapping the 256-dimensional input to a 50-dimensional bottleneck and back to 256 dimensions. ReLU = rectified linear unit activation function; Sigmoid = logistic activation function.

50, which was chosen due to its smallest generalization gap (see Table 9).

The generalization gap refers to the difference between a model's performance on training data versus unseen data. In NM, we want detected deviations to reflect meaningful pathological differences rather than artifacts of poor generalization. If a model has a large generalization gap, deviations in symptomatic subjects may be indistinguishable from errors due to poor generalization. By demonstrating that our model does not exhibit significantly larger errors on unseen HC (test set) than on seen HC (train and validation set), we increase the likelihood that deviations observed in symptomatic subjects truly reflect clinically meaningful effects. To confirm the absence of such effects, we compared reconstruction error distributions between training, validation, and test subsets of HCs using one-sided Mann–Whitney U tests. We found no significant differences between training and validation ($p = 0.26$) or between training and test ($p = 0.50$) reconstruction error distribution, supporting the stability and generalizability of our model.

2.4 Shift Analysis

To quantify how diagnostic groups deviate from the natural variability present in HCs, we performed a shift analysis based on the full distribution of deviation scores. This approach moves beyond mean- or median-based comparisons by assessing how the *entire shape* of the deviation distribution in symptomatic groups diverges from that of HCs. What follows is a mathematical description of the steps performed to quantify the *shift*:

1. Mahalanobis Distance Computation For each subject, we computed the deviation from the normative reference distribution derived from HCs in the deconfounded embedding space. These distances reflect how strongly an individual's brain representation deviates from the normative manifold. After this step, we performed the group-wise significance testing described in the manuscript to ensure meaningfulness of the deviations.

2. Kernel Density Estimation For each diagnosis, we extracted the full set of deviations for all symptomatic groups and compared them to the HC distribution using Gaussian kernel density estimation (KDE) with Silverman's rule for bandwidth selection [8]. This procedure yields smooth estimates of the probability density functions for deviations in both HCs and symptomatic subjects.

3. Exponential Weighting of Deviations To prioritize clinically relevant patterns, we applied an exponential weighting function over the deviation axis, defined as:

$$w(x) = \exp\left(\frac{x}{\max(x)}\right),$$

where x denotes the deviation. This weighting emphasizes larger deviations, under the assumption that they are more likely to reflect pathological alterations rather than normative variability, and we want the shift to be higher if it appears in high deviation density regions.

4. Normalized Excess Area We quantified the *weighted excess area*—the region where the symptomatic group density exceeds the HC density—according to:

$$\text{Excess Area} = \int_{x:\, f_{\text{symptomatic}}(x) > f_{\text{HC}}(x)} \left(f_{\text{symptomatic}}(x) - f_{\text{HC}}(x)\right) w(x)\, dx,$$

where $f_{\text{symptomatic}}(x)$ and $f_{\text{HC}}(x)$ are the KDEs of symptomatic and HC deviations, respectively, and $w(x)$ is the exponential weighting function.

The total weighted area under the symptomatic subject curve normalized this value:

$$\text{Normalization} = \int f_{\text{symptomatic}}(x) \cdot w(x)\, dx,$$

and the final metric was computed as:

$$\text{Fraction of Unexplained Abnormality ("shift")} = \frac{\text{Excess Area}}{\text{Normalization}}.$$

This yields the final easy-to-interpret shift, emphasizing large deviations more harshly.

2.5 Normative Model Stability

To evaluate the robustness of the estimated normative reference distribution parameters (mean μ, covariance Σ), we conducted a bootstrap-based stability analysis.

Specifically, we resampled the full set of HC deviations with replacement 1,000 times, computing the mean vector and covariance matrix for each bootstrap sample. To assess variability, we used multiple stability metrics: (i) the average Euclidean distance and cosine similarity between

bootstrap means and their overall centroid, and (ii) the average Frobenius norm between bootstrap covariance matrices and the mean covariance matrix. The Frobenius norm is defined as:

$$\|\Sigma\|_F = \sqrt{\sum_{i=1}^{d}\sum_{j=1}^{d} |a_{ij}|^2} \tag{1}$$

where $\Sigma \in \mathbb{R}^{d\times d}$ is the covariance matrix parameter of the Mahalanobis distance. This analysis was performed separately for both the NAKO and UKB cohorts.

In the NAKO cohort, results indicated low variability: the mean Euclidean distance between bootstrapped means was 0.0019, the cosine similarity was 0.9995, and the normalized covariance stability score was 0.0475 (with zero indicating perfect stability). The UKB cohort exhibited even greater stability, with a mean distance of 0.0002, a cosine similarity of 0.99998, and a normalized covariance score of 0.017. Covariance stability was defined as the average Frobenius norm between each bootstrap covariance and the mean covariance, normalized by the Frobenius norm of the mean covariance. This yields a scale-invariant measure of how consistently the covariance structure is preserved across resampled subsets, with lower values reflecting greater stability.

These findings confirm that the normative model Mahalanobis parameters (mean and covariance) are highly stable across bootstrap samples in both cohorts, supporting the reliability of the learned HC deviation distributions.

2.6 Directional Analysis

Algorithm 1

Require:

Patient table with embeddings $X_p \in \mathbb{R}^{n_p \times D}$, confounders $C_p \in \mathbb{R}^{n_p \times q}$, and group indicators $\{g(i)\}_{i=1}^{n_p}$ over label set $\mathcal{G}$

HC table (already joined) with embeddings $X_h \in \mathbb{R}^{n_h \times D}$ and confounders $C_h \in \mathbb{R}^{n_h \times q}$

1: Concatenate $X \leftarrow \begin{bmatrix} X_p \\ X_h \end{bmatrix} \in \mathbb{R}^{N \times D}$, $C \leftarrow \begin{bmatrix} C_p \\ C_h \end{bmatrix} \in \mathbb{R}^{N \times q}$ where $N = n_p + n_h$.

2: **Linear residualization.** For each feature $j = 1, \ldots, D$, solve

$$(\hat{\alpha}_j, \hat{\beta}_j) \in \arg\min_{\alpha,\beta} \ \left\| X_{\cdot j} - \alpha \mathbf{1} - C\beta \right\|_2^2, \quad R_{\cdot j} \leftarrow X_{\cdot j} - \hat{\alpha}_j \mathbf{1} - C\hat{\beta}_j.$$

Let $R \in \mathbb{R}^{N \times D}$ collect residuals.

3: **Standardize.** For $j = 1, \ldots, D$ let $\mu_j = \frac{1}{N} \sum_{i=1}^{N} R_{ij}$ and $\sigma_j^2 = \frac{1}{N} \sum_{i=1}^{N} (R_{ij} - \mu_j)^2$. Set $\tilde{R}_{ij} = (R_{ij} - \mu_j)/\sigma_j$; denote $\tilde{R} \in \mathbb{R}^{N \times D}$.

4: **PCA to 2D.** Form $\Sigma = \frac{1}{N} \tilde{R}^\top \tilde{R}$ and take top-2 eigenvectors $W \in \mathbb{R}^{D \times 2}$. Project $Z = \tilde{R}W \in \mathbb{R}^{N \times 2}$ and split $Z = [Z_p; Z_h]$.

5: **HC robust center.** Compute $(c_h, \Lambda_h, Q_h) = \textsc{BootstrapEllipse}(Z_h, B)$ (Alg. 2).

6: **Recentering.** Translate $Z \leftarrow Z - \mathbf{1}c_h^\top$; obtain Z_p, Z_h now centered at the HC median.

7: **Group ellipses.** For each label $g \in \mathcal{G}$, define $S_g = \{z_i \in Z_p : \ g(i) = g\}$. If $|S_g| > 0$, compute $(c_g, \Lambda_g, Q_g) = \textsc{BootstrapEllipse}(S_g, B)$.

8: **Ellipse parameters.** Each ellipse is represented by center $c \in \mathbb{R}^2$ and covariance-like shape $\Sigma^\star = Q\Lambda Q^\top$ where $\Lambda = \mathrm{diag}(\lambda_1, \lambda_2)$. The principal axes are Qe_1, Qe_2 with semi-axis lengths $a_k = \sqrt{\lambda_k}$.

Algorithm 2 BootstrapEllipse: robust ellipse from geometric-median bootstraps

---

Require: Point set $S = \{x_i\}_{i=1}^n \subset \mathbb{R}^2$, bootstrap count B

1: **for** $b = 1$ to B **do**

2: Draw a bootstrap sample $S^{(b)}$ by sampling n points with replacement from S

3: Compute bootstrap geometric median

$$m_b \in \arg\min_{m \in \mathbb{R}^2} \sum_{x \in S^{(b)}} \|x - m\|_2 \quad \text{(e.g. via Weiszfeld's algorithm [9])}$$

4: **end for**

5: Form $M = \{m_b\}_{b=1}^B$ and its geometric median $c \in \arg\min_m \sum_{b=1}^B \|m_b - m\|_2$ (robust center).

6: Compute the covariance of $\{m_b\}$ with a small ridge: $\Sigma = \mathrm{Cov}(M) + \lambda I_2$ (e.g. $\lambda = 10^{-9}$).

7: Eigendecompose $\Sigma = Q\,\mathrm{diag}(\lambda_1, \lambda_2)\,Q^\top$ with $\lambda_1 \geq \lambda_2 \geq 0$.

8: **return** c (center), $\Lambda = \mathrm{diag}(\lambda_1, \lambda_2)$, and Q (principal directions).

2.7 UKB External Validation Setup Details

In addition to transferring the models, we also transfer the deconfounding parameters for all confounders except the data collection center, as this variable reflects a dataset-specific effect. To address center-specific differences, we retrain the linear deconfounder for the center variable using only UKB data. For consistency in feature scaling, we apply the same min–max normalization used for the deconfounded embeddings. Due to cohort-specific effects, we had to re-estimate the Mahalanobis distance parameters, μ and Σ, from the HCs within the UKB cohort rather than transferring them from NAKO, because the original parameters yielded unrealistically high Mahalanobis distances in the UKB data (median 44.24 vs. 11.93 when using the NAKO parameters).

For estimating the Mahalanobis parameters used in the shift analysis and significance testing, we applied the same procedure as in NAKO. Specifically, we split the HC data into 90% training and 10% testing. The mean vector μ and covariance matrix Σ were estimated on the 90% training set, and the shift analysis and significance testing were conducted on the remaining 10%. As detailed in Section 2.5, the Mahalanobis parameters in the UKB were found to be robust to sampling variability based on extensive bootstrapping, indicating that these parameters are highly stable within each cohort and largely insensitive to the specific subset of healthy controls used for fitting.

Finally, we reuse the PCA parameters fitted on the NAKO dataset for the directional deviation analysis in UKB.

3 Extended Results

3.1 Autoencoder Dimensionality Tuning

Table 9: Evaluation of autoencoder bottleneck dimensionality. Models were trained with different bottleneck sizes and evaluated across five random seeds. Reported values are mean and standard deviation of validation reconstruction error (MSE) and downstream classification performance.

Bottleneck	Val. MSE Mean	Val. SD
10	0.005560	0.000457
25	0.005228	0.001773
50	0.003839	0.000318
75	0.007397	0.003850
100	0.005952	0.003095

3.2 Deconfounding

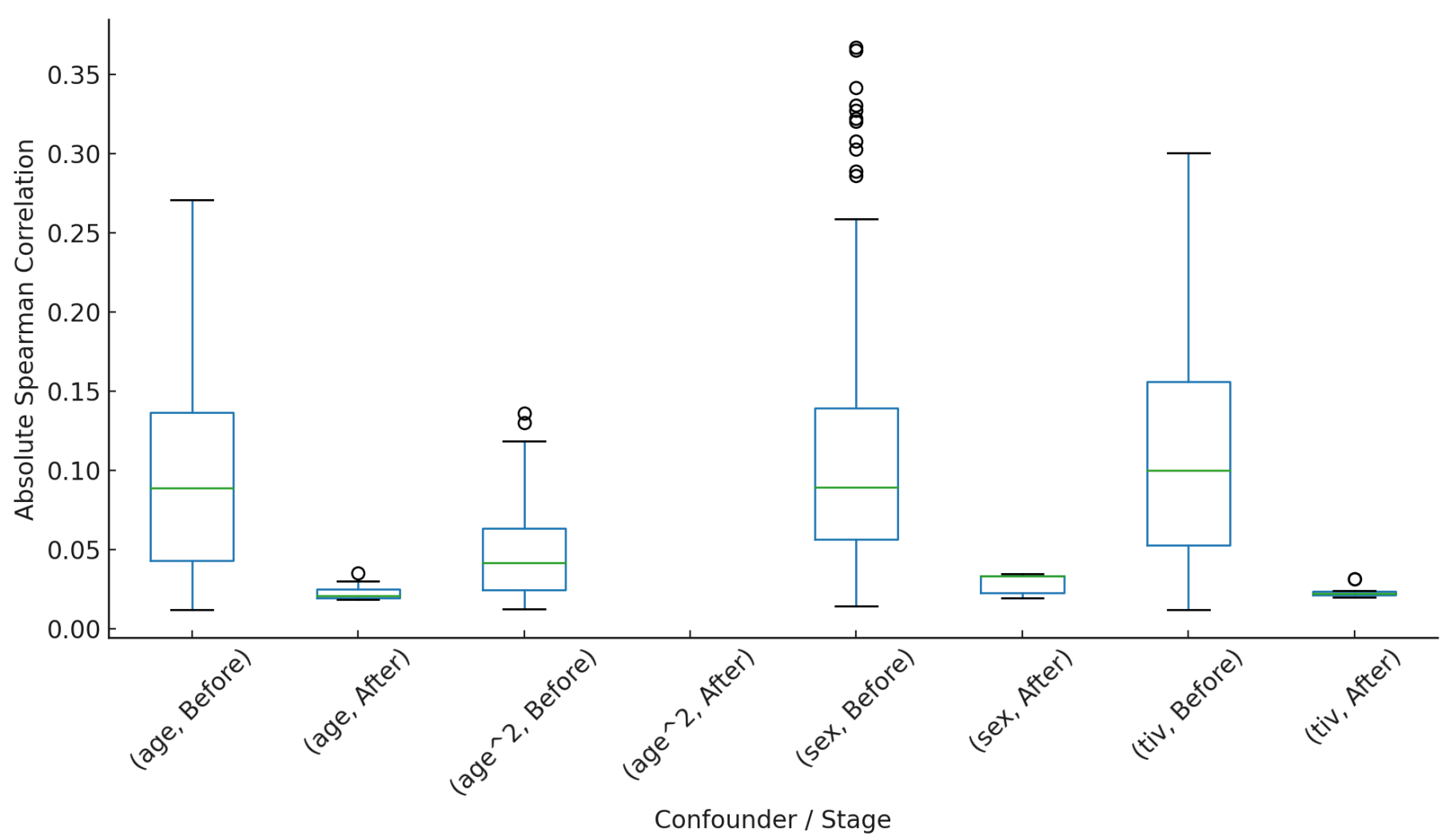

Figure 2: Distributions of absolute Spearman rank correlation coefficients between embedding dimensions and confounders before and after deconfounding. Confounders include age, age-squared, sex, and total intracranial volume (TIV). Only correlations significant at $p_{\mathrm{FDR}} < 0.05$ after Benjamini–Hochberg correction are shown.

3.3 Shift Analysis Extended Results

Figure 3 presents the shift analysis using self-reported doctors' diagnoses and MINI-based diagnoses for NAKO, and ICD-10-based diagnoses for UKB.

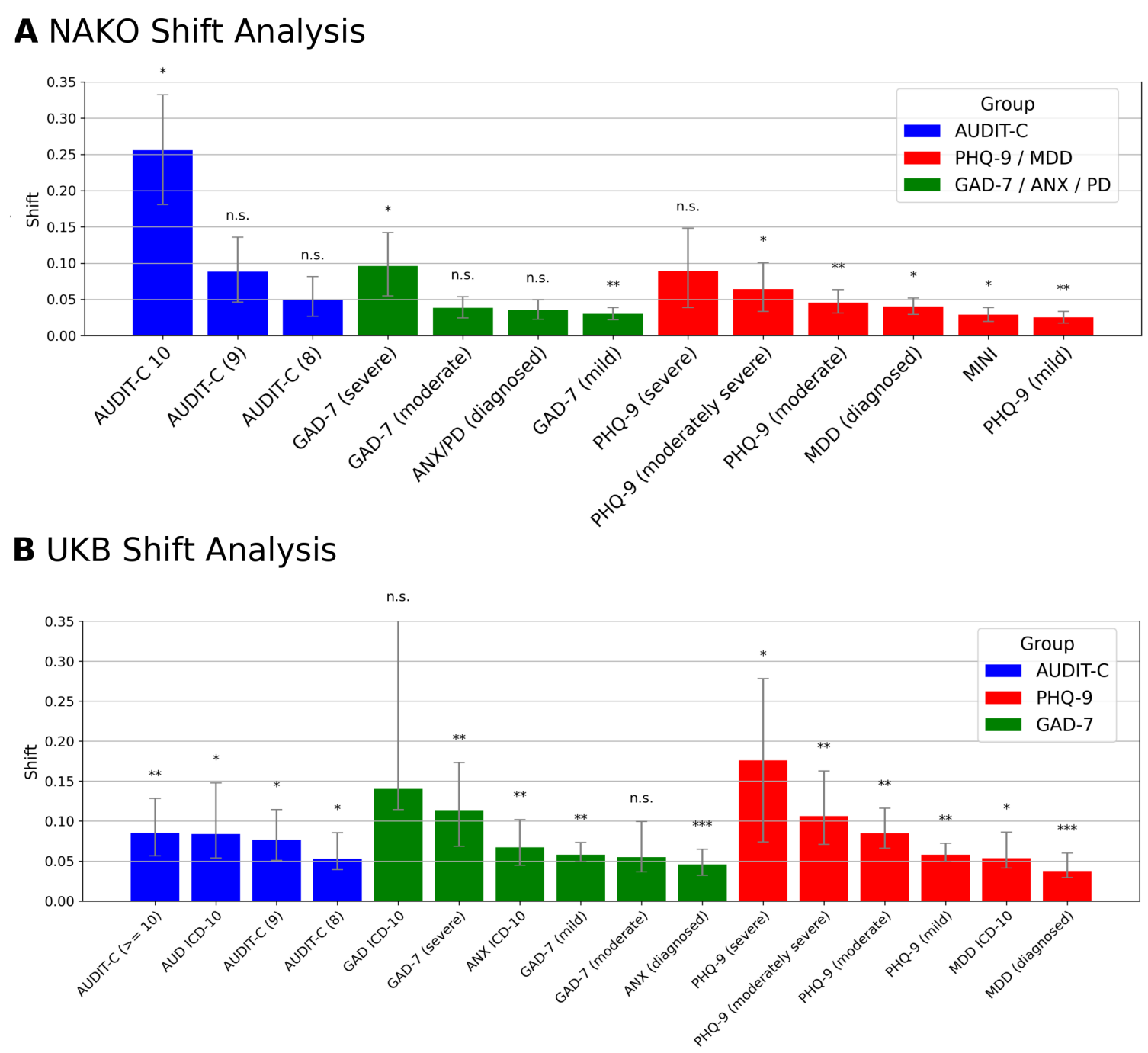

Figure 3: Fraction of group deviations unexplained by healthy control (HC) variability ("shift") for all symptomatic groups in the NAKO cohort (German National Cohort) (**A**) and the UKB cohort (UK Biobank) (**B**). Deviation scores were modeled using linear regression with group membership as the main predictor, adjusting for sex, age, age-squared, and sex–age interactions. Symptomatic groups were defined by AUDIT-C (Alcohol Use Disorders Identification Test–Consumption), PHQ-9 (Patient Health Questionnaire-9) for major depressive disorder (MDD), GAD-7 (Generalized Anxiety Disorder-7), and self-reported physician- or hospital record ICD-10–based diagnoses of anxiety disorders (ANX/PD) or MDD. Asterisks indicate significance levels after Benjamini–Hochberg false discovery rate (FDR) correction: * $p_{\mathrm{FDR}} < 0.05$, ** $p_{\mathrm{FDR}} < 0.01$, *** $p_{\mathrm{FDR}} < 0.005$.

Figure 4 presents the shift analysis in NAKO separated by sex.

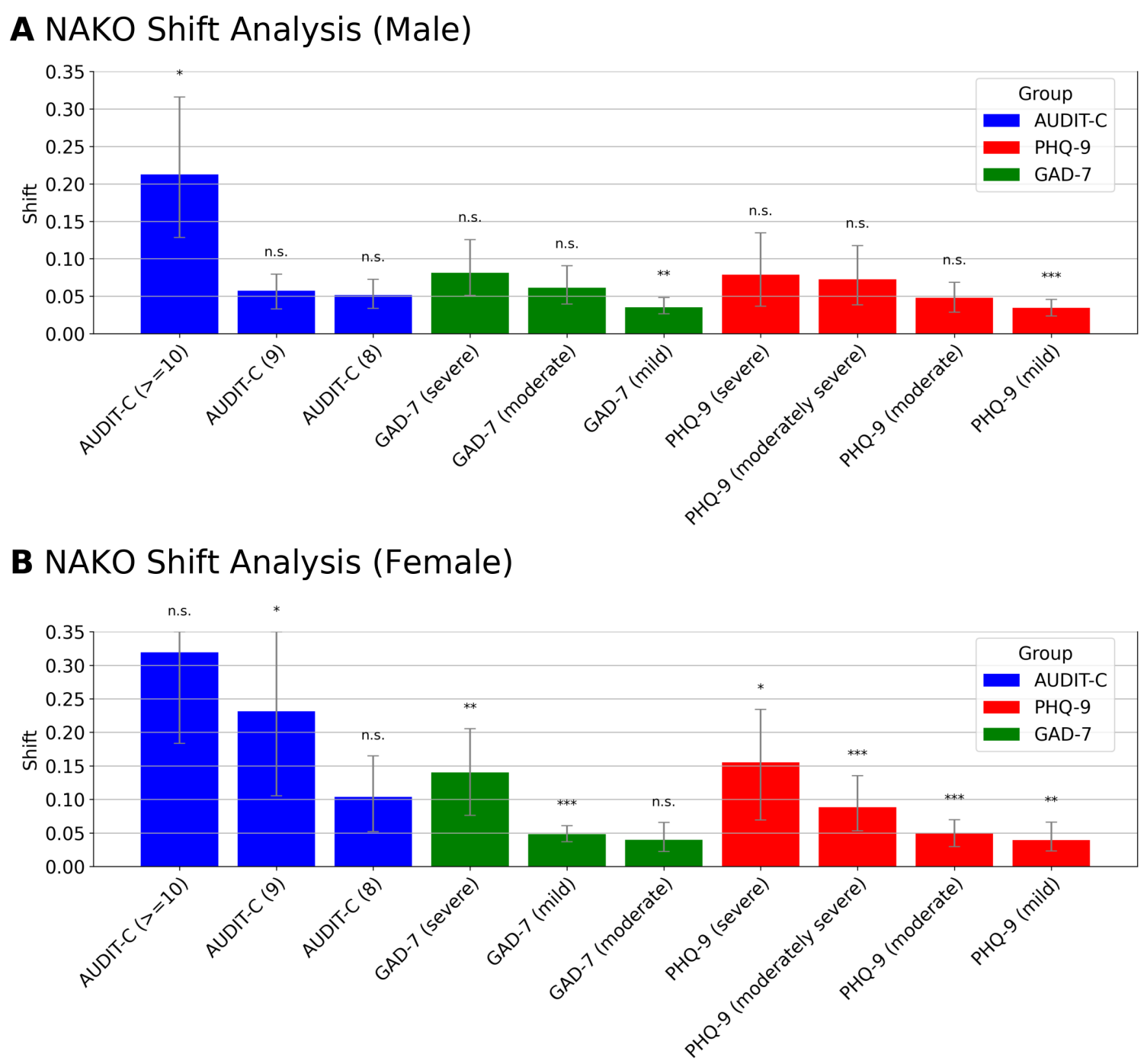

Figure 4: Fraction of group deviations unexplained by healthy control (HC) variability ("shift") in the NAKO cohort (German National Cohort), stratified by sex: (**A**) males and (**B**) females. Deviation scores were modeled using linear regression with group membership as the main predictor, adjusting for age and age-squared. Symptomatic groups were defined by AUDIT-C (Alcohol Use Disorders Identification Test–Consumption), PHQ-9 (Patient Health Questionnaire-9), and GAD-7 (Generalized Anxiety Disorder-7) thresholds. Asterisks indicate significance levels after Benjamini–Hochberg false discovery rate (FDR) correction: * $p_{\mathrm{FDR}} < 0.05$, ** $p_{\mathrm{FDR}} < 0.01$, *** $p_{\mathrm{FDR}} < 0.005$.

**Diagnosis**	**p-value**	$\mathbf{p}_{\mathrm{FDR}}$
MINI	0.0080	0.0174
MDD (diagnosed)	0.0070	0.0174
ANX/PD (diagnosed)	0.2517	0.2975
AUDIT-C (8)	0.7128	0.7128
AUDIT-C (9)	0.0857	0.1237
AUDIT-C (>=10)	0.0045	0.0147
PHQ-9 (mild)	0.0016	0.0088
PHQ-9 (moderate)	0.0008	0.0088
PHQ-9 (moderately severe)	0.0143	0.0265
PHQ-9 (severe)	0.1537	0.1998
GAD-7 (mild)	0.0020	0.0088
GAD-7 (moderate)	0.4490	0.4864
GAD-7 (severe)	0.0258	0.0419

Table 10: ANOVA results for the NAKO cohort (German National Cohort), reporting raw p-values (test of the null hypothesis that group means are equal, PR($> F$)) and Benjamini–Hochberg false discovery rate (FDR)–adjusted p-values (p_{FDR}) for each diagnostic group. MINI = Mini International Neuropsychiatric Interview; MDD = major depressive disorder; ANX/PD = anxiety or panic disorder (doctor's diagnosis); AUDIT-C = Alcohol Use Disorders Identification Test–Consumption; PHQ-9 = Patient Health Questionnaire-9; GAD-7 = Generalized Anxiety Disorder-7.

**Tested Group**	**p-value**	$\mathbf{p}_{\mathrm{FDR}}$
MDD (ICD-10)	0.0153	0.0231
AUD (ICD-10)	0.0396	0.0453
AUDIT-C (8)	0.0391	0.0453
AUDIT-C (9)	0.0173	0.0231
AUDIT-C (>=10)	0.0054	0.0096
ANX (ICD-10)	0.0025	0.0071
GAD (ICD-10)	0.2325	0.2874
PHQ-9 (mild)	0.0031	0.0071
PHQ-9 (moderate)	0.0010	0.0052
PHQ-9 (moderately severe)	0.0025	0.0071
PHQ-9 (severe)	0.0166	0.0231
GAD-7 (mild)	0.0031	0.0071
GAD-7 (moderate)	0.1723	0.1838
GAD-7 (severe)	0.0052	0.0096
MDD (diagnosed)	0.0002	0.0015
ANX (diagnosed)	0.00005	0.0008

Table 11: ANOVA results for the UKB cohort (UK Biobank), reporting raw p-values (test of the null hypothesis that group means are equal, PR($> F$)) and Benjamini–Hochberg false discovery rate (FDR)–adjusted p-values (p_{FDR}) for each tested group. MDD = major depressive disorder; AUD = alcohol use disorder; ANX = anxiety disorder; GAD = generalized anxiety disorder; PHQ-9 = Patient Health Questionnaire-9; GAD-7 = Generalized Anxiety Disorder-7.

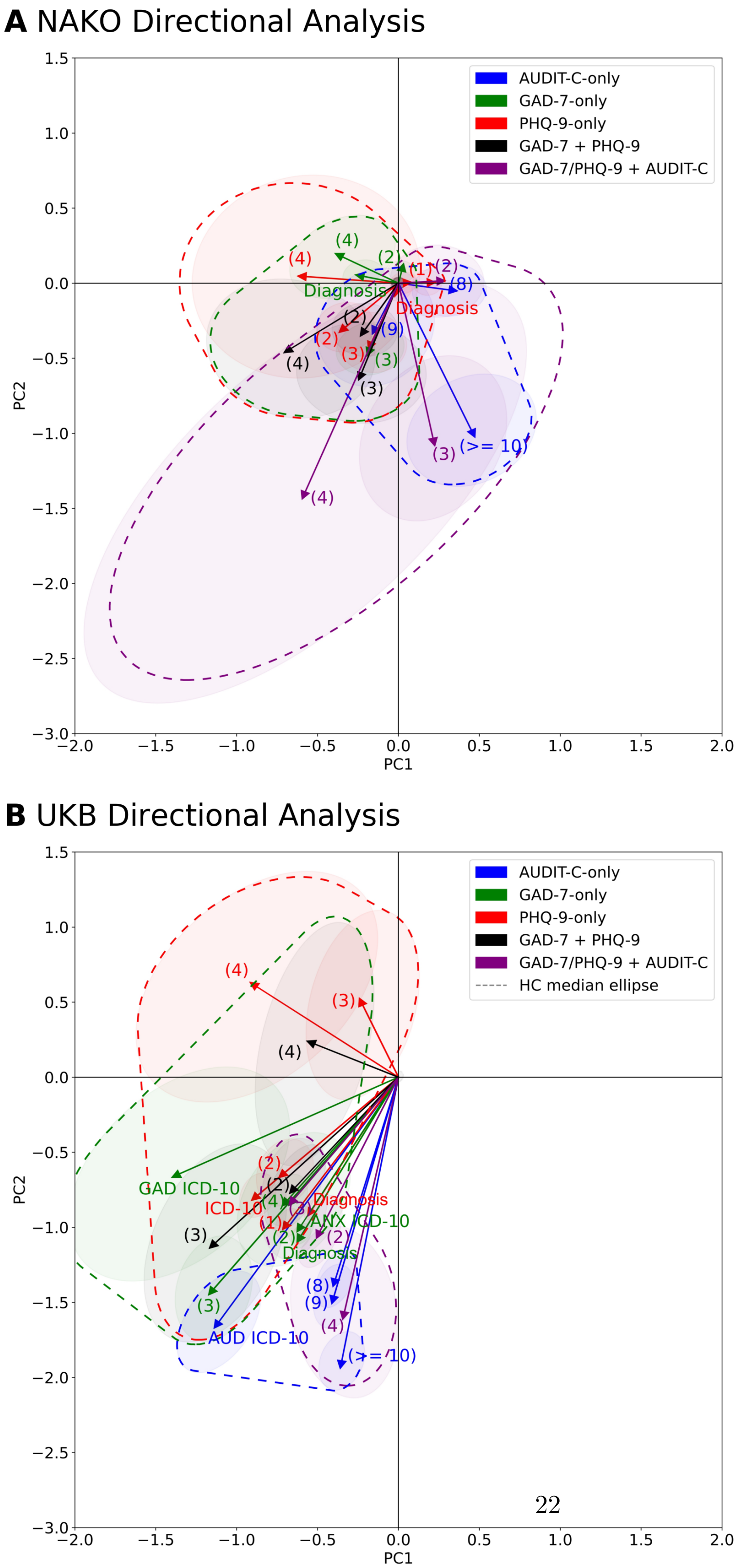

Figure 5: Directional analyses of normative deviation vectors based on principal component analysis (PCA; two dimensions, 21% explained variance) for diagnostic groups in the NAKO (German National Cohort) (**A**) and UKB (UK Biobank) cohort (**B**). Shaded ellipses show the ±1 SD contour of the bootstrap (1,000 resamples) distribution around the group-level geometric median vectors. The dark ellipse at the origin represents the median deviation among healthy controls (HCs), serving as a reference for normative variability. Numbers indicate severity levels: PHQ-9 (Patient Health Questionnaire-9) levels (1–4) = mild, moderate, moderately severe, severe; GAD-7 (Generalized Anxiety Disorder-7) levels (2–4) = mild, moderate, severe; AUDIT-C (Alcohol Use Disorders Identification Test–Consumption) = symptom count. "MDD/GAD + ALC" denotes individuals with acute major depressive disorder (MDD) or generalized anxiety disorder (GAD) symptoms and elevated alcohol use (AUDIT-C $\geq$ 10). "ICD-10" refers to International Classification of Diseases, 10th Revision, hospital record codes, and "Diagnosis" to self-reported conditions. Red ellipses = PHQ/GAD groups, blue = AUDIT-C groups, purple = comorbid groups.

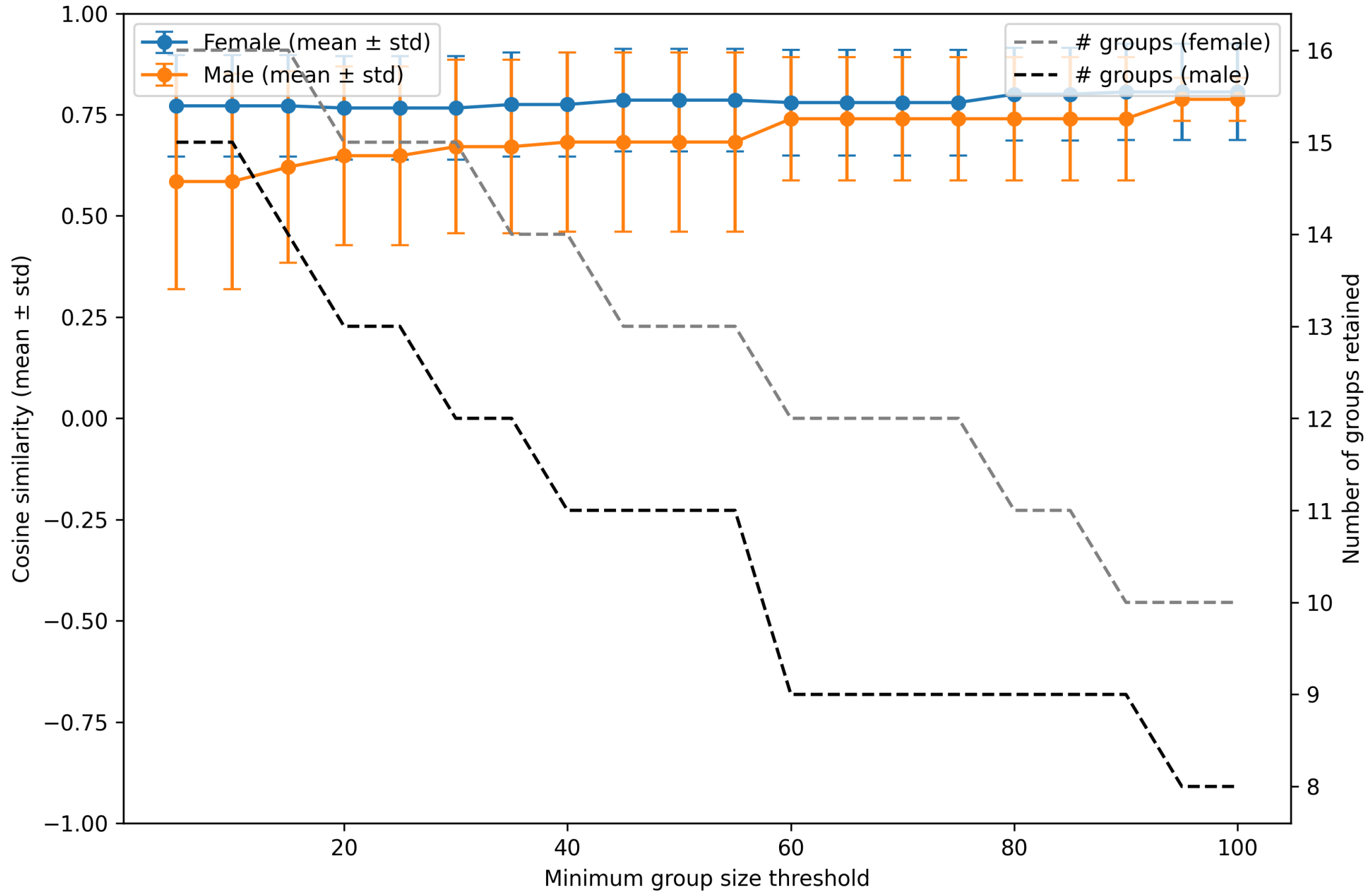

Figure 6: Cosine similarity analysis results for directional analysis with sex stratification. Error bars denote standard deviations across groups; similarity is computed to the joint-sex median vector. The x-axis indicates the minimum group size required for inclusion in the analysis. Dashed lines show the number of groups meeting this threshold.

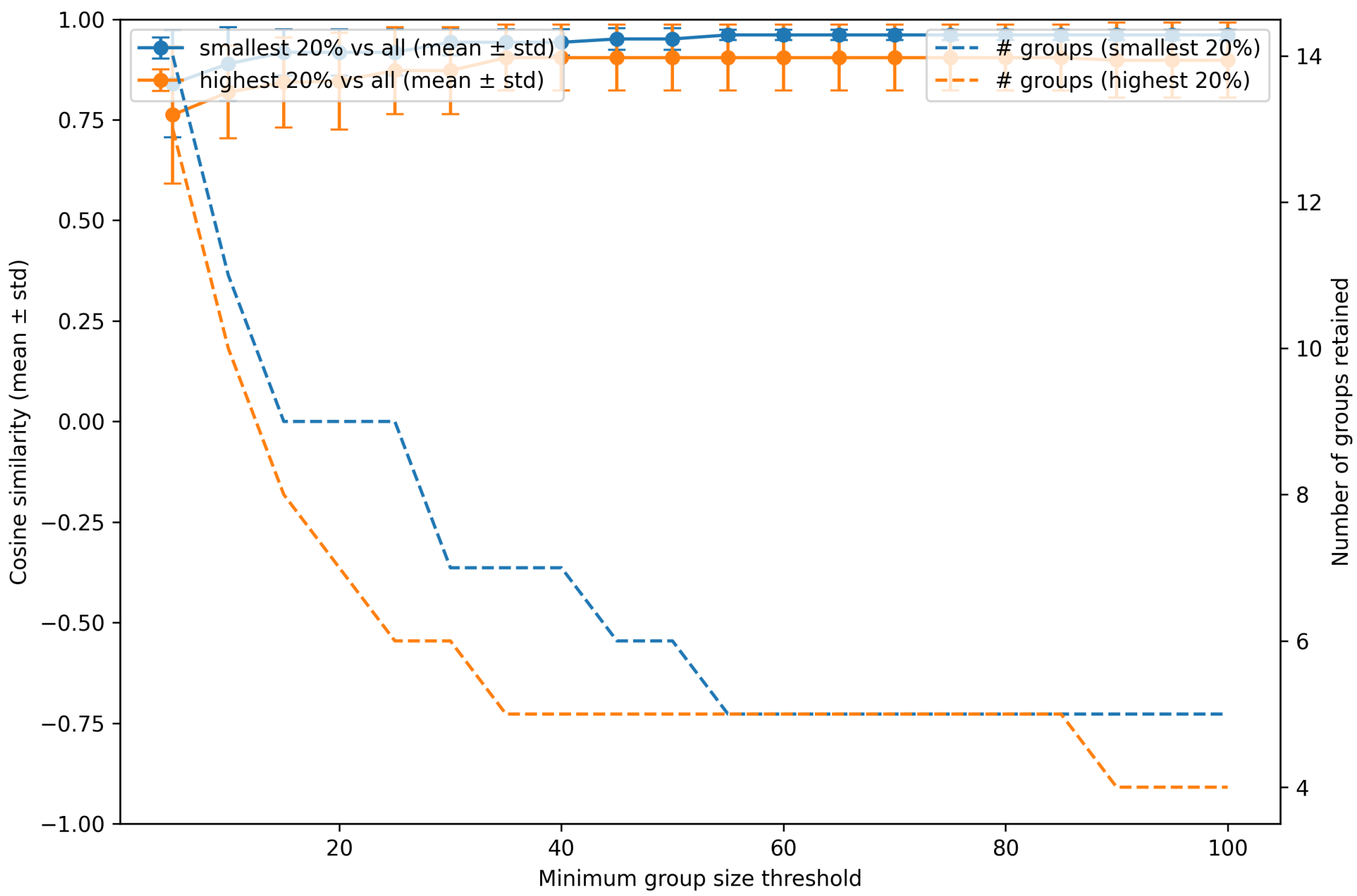

Figure 7: Influence of the interval between MRI and questionnaire acquisition. Error bars denote the standard deviation across groups. Cosine similarity (y-axis) reflects the similarity of each group's median vector (bottom 20% and top 20% of the interval distribution) to the unfiltered median vector. The x-axis indicates the minimum group size required for inclusion in the analysis. Dashed lines show the number of groups meeting this threshold.

3.4 List of FreeSurfer Measures

Table 12: List of Freesurfer measures used for deviation association analysis and classification baseline

**FreeSurfer Measure**
aseg.volume_Left-Cerebellum-Cortex
aseg.volume_Left-Thalamus
aseg.volume_Left-Caudate
aseg.volume_Left-Putamen
aseg.volume_Left-Pallidum
aseg.volume_Left-Hippocampus
aseg.volume_Left-Amygdala
aseg.volume_Left-Accumbens-area
aseg.volume_Left-VentralDC
aseg.volume_Right-Cerebellum-Cortex
aseg.volume_Right-Thalamus
aseg.volume_Right-Caudate
aseg.volume_Right-Putamen
aseg.volume_Right-Pallidum
aseg.volume_Right-Hippocampus
aseg.volume_Right-Amygdala
aseg.volume_Right-Accumbens-area
aseg.volume_Right-VentralDC
aseg.volume_non-WM-hypointensities
aseg.volume_Left-non-WM-hypointensities
aseg.volume_Right-non-WM-hypointensities
aseg.volume_BrainSegVol
aseg.volume_BrainSegVolNotVent
aseg.volume_lhCortexVol
aseg.volume_rhCortexVol
aseg.volume_CortexVol
aseg.volume_SubCortGrayVol
aseg.volume_TotalGrayVol
aseg.volume_SupraTentorialVol
aseg.volume_SupraTentorialVolNotVent
aseg.volume_EstimatedTotalIntraCranialVol
lh.aparc.volume_lh_bankssts_volume
lh.aparc.volume_lh_caudalanteriorcingulate_volume
lh.aparc.volume_lh_caudalmiddlefrontal_volume
lh.aparc.volume_lh_cuneus_volume
lh.aparc.volume_lh_entorhinal_volume
lh.aparc.volume_lh_fusiform_volume
lh.aparc.volume_lh_inferiorparietal_volume
lh.aparc.volume_lh_inferiortemporal_volume
lh.aparc.volume_lh_isthmuscingulate_volume

**FreeSurfer Measure (continued)**
lh.aparc.volume_lh_lateraloccipital_volume
lh.aparc.volume_lh_lateralorbitofrontal_volume
lh.aparc.volume_lh_lingual_volume
lh.aparc.volume_lh_medialorbitofrontal_volume
lh.aparc.volume_lh_middletemporal_volume
lh.aparc.volume_lh_parahippocampal_volume
lh.aparc.volume_lh_paracentral_volume
lh.aparc.volume_lh_parsopercularis_volume
lh.aparc.volume_lh_parsorbitalis_volume
lh.aparc.volume_lh_parstriangularis_volume
lh.aparc.volume_lh_pericalcarine_volume
lh.aparc.volume_lh_postcentral_volume
lh.aparc.volume_lh_posteriorcingulate_volume
lh.aparc.volume_lh_precentral_volume
lh.aparc.volume_lh_precuneus_volume
lh.aparc.volume_lh_rostralanteriorcingulate_volume
lh.aparc.volume_lh_rostralmiddlefrontal_volume
lh.aparc.volume_lh_superiorfrontal_volume
lh.aparc.volume_lh_superiorparietal_volume
lh.aparc.volume_lh_superiortemporal_volume
lh.aparc.volume_lh_supramarginal_volume
lh.aparc.volume_lh_frontalpole_volume
lh.aparc.volume_lh_temporalpole_volume
lh.aparc.volume_lh_transversetemporal_volume
lh.aparc.volume_lh_insula_volume
rh.aparc.volume_rh_bankssts_volume
rh.aparc.volume_rh_caudalanteriorcingulate_volume
rh.aparc.volume_rh_caudalmiddlefrontal_volume
rh.aparc.volume_rh_cuneus_volume
rh.aparc.volume_rh_entorhinal_volume
rh.aparc.volume_rh_fusiform_volume
rh.aparc.volume_rh_inferiorparietal_volume
rh.aparc.volume_rh_inferiortemporal_volume
rh.aparc.volume_rh_isthmuscingulate_volume
rh.aparc.volume_rh_lateraloccipital_volume
rh.aparc.volume_rh_lateralorbitofrontal_volume
rh.aparc.volume_rh_lingual_volume
rh.aparc.volume_rh_medialorbitofrontal_volume
rh.aparc.volume_rh_middletemporal_volume
rh.aparc.volume_rh_parahippocampal_volume
rh.aparc.volume_rh_paracentral_volume
rh.aparc.volume_rh_parsopercularis_volume
rh.aparc.volume_rh_parsorbitalis_volume
rh.aparc.volume_rh_parstriangularis_volume
rh.aparc.volume_rh_pericalcarine_volume

FreeSurfer Measure (continued)
rh.aparc.volume_rh_postcentral_volume
rh.aparc.volume_rh_posteriorcingulate_volume
rh.aparc.volume_rh_precentral_volume
rh.aparc.volume_rh_precuneus_volume
rh.aparc.volume_rh_rostralanteriorcingulate_volume
rh.aparc.volume_rh_rostralmiddlefrontal_volume
rh.aparc.volume_rh_superiorfrontal_volume
rh.aparc.volume_rh_superiorparietal_volume
rh.aparc.volume_rh_superiortemporal_volume
rh.aparc.volume_rh_supramarginal_volume
rh.aparc.volume_rh_frontalpole_volume
rh.aparc.volume_rh_temporalpole_volume
rh.aparc.volume_rh_transversetemporal_volume
rh.aparc.volume_rh_insula_volume

3.5 Correlation Analysis

Table 13: Significant Spearman rank correlations between latent embedding dimensions and symptom severity scores after false discovery rate (FDR) correction ($q < 0.05$). The estimated proportion of true null hypotheses (π_0), derived using the q-value procedure [10], was 0.74. For each association, Spearman's r, uncorrected p-values, and corresponding FDR-adjusted q-values are reported.

Score	Dimension	Spearman r	q_{value}	$p_{\text{value\_uncorrected}}$
AUDIT-C sum score	48	-0.027	1.135e-03	3.738e-06
AUDIT-C sum score	68	-0.027	1.135e-03	4.027e-06
AUDIT-C sum score	221	0.026	1.982e-03	1.055e-05
AUDIT-C sum score	20	-0.025	2.174e-03	1.691e-05
AUDIT-C sum score	136	0.025	2.174e-03	1.929e-05
AUDIT-C sum score	13	-0.024	3.245e-03	3.582e-05
AUDIT-C sum score	142	0.024	3.245e-03	4.605e-05
AUDIT-C sum score	145	-0.024	3.245e-03	4.563e-05
AUDIT-C sum score	114	0.024	3.498e-03	5.585e-05
AUDIT-C sum score	213	0.024	3.793e-03	6.729e-05
AUDIT-C sum score	16	-0.023	3.817e-03	7.449e-05
AUDIT-C sum score	217	0.023	5.072e-03	1.260e-04
AUDIT-C sum score	207	0.022	7.509e-03	2.132e-04
AUDIT-C sum score	150	0.021	9.336e-03	2.982e-04
AUDIT-C sum score	227	-0.021	9.336e-03	2.837e-04
AUDIT-C sum score	52	0.021	9.996e-03	3.370e-04
AUDIT-C sum score	100	0.021	1.120e-02	4.174e-04

Continued on next page

Table 13: Significant Spearman rank correlations between latent embedding dimensions and symptom severity scores after false discovery rate (FDR) correction ($q < 0.05$). The estimated proportion of true null hypotheses (π_0), derived using the q-value procedure [10], was 0.74. For each association, Spearman's r, uncorrected p-values, and corresponding FDR-adjusted q-values are reported.

Score	Dimension	Spearman r	q_{value}	$p_{\text{value\_uncorrected}}$
AUDIT-C sum score	224	-0.021	1.120e-02	4.165e-04
AUDIT-C sum score	162	0.021	1.135e-02	4.553e-04
AUDIT-C sum score	204	-0.021	1.135e-02	4.631e-04
AUDIT-C sum score	155	0.020	1.155e-02	5.326e-04
AUDIT-C sum score	86	-0.020	1.268e-02	6.074e-04
AUDIT-C sum score	73	0.020	1.386e-02	6.884e-04
AUDIT-C sum score	62	0.019	2.478e-02	1.599e-03
AUDIT-C sum score	87	0.019	2.478e-02	1.528e-03
AUDIT-C sum score	122	0.019	2.478e-02	1.581e-03
AUDIT-C sum score	216	-0.019	2.478e-02	1.533e-03
AUDIT-C sum score	182	0.018	2.900e-02	1.955e-03
AUDIT-C sum score	102	0.018	3.010e-02	2.083e-03
AUDIT-C sum score	112	0.018	3.104e-02	2.238e-03
AUDIT-C sum score	223	-0.017	3.931e-02	3.208e-03
AUDIT-C sum score	31	-0.017	4.784e-02	4.244e-03
AUDIT-C sum score	177	-0.017	4.784e-02	4.161e-03
AUDIT-C sum score	61	0.017	4.888e-02	4.509e-03
AUDIT-C sum score	173	0.017	4.901e-02	4.609e-03
GAD-7 sum score	100	-0.019	2.478e-02	1.306e-03
GAD-7 sum score	241	0.019	2.478e-02	1.445e-03
GAD-7 sum score	217	-0.017	3.931e-02	3.087e-03
GAD-7 sum score	58	0.017	4.821e-02	4.362e-03
PHQ-9 sum score	76	0.023	4.865e-03	1.069e-04
PHQ-9 sum score	217	-0.023	4.865e-03	1.122e-04
PHQ-9 sum score	246	0.022	6.592e-03	1.754e-04
PHQ-9 sum score	100	-0.021	1.155e-02	4.924e-04
PHQ-9 sum score	205	0.020	1.155e-02	5.170e-04
PHQ-9 sum score	132	-0.019	2.478e-02	1.626e-03
PHQ-9 sum score	225	0.019	2.478e-02	1.418e-03
PHQ-9 sum score	231	0.019	2.478e-02	1.606e-03
PHQ-9 sum score	6	-0.018	3.104e-02	2.313e-03
PHQ-9 sum score	233	0.018	3.104e-02	2.267e-03
PHQ-9 sum score	45	-0.017	3.931e-02	3.203e-03
PHQ-9 sum score	91	-0.017	3.931e-02	3.126e-03
PHQ-9 sum score	227	0.017	4.161e-02	3.470e-03
PHQ-9 sum score	216	-0.017	4.784e-02	4.222e-03

3.6 z-Scored Symptom Score Mapping Brains

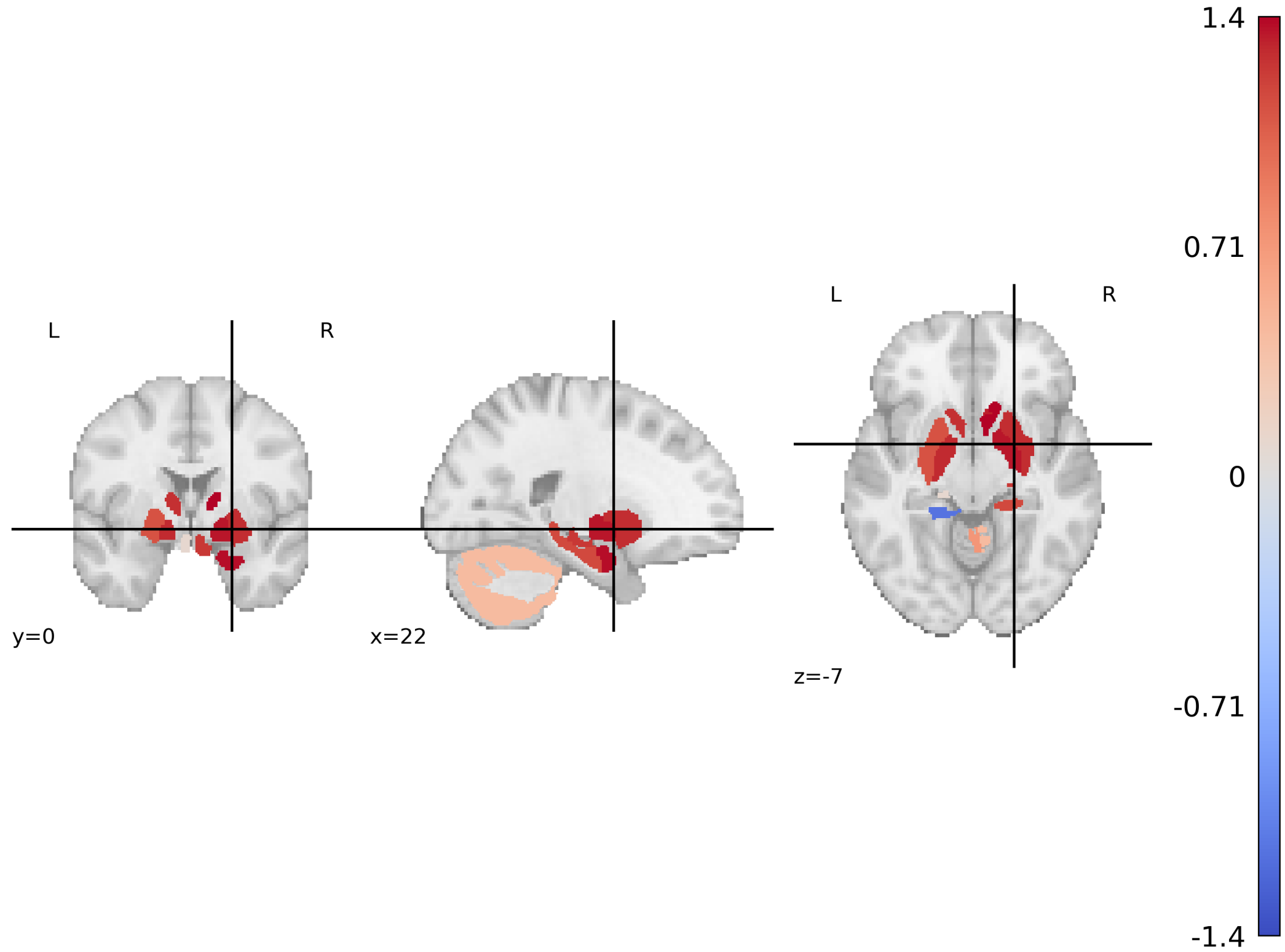

Figure 8: Z-scored heuristic associations between latent embedding dimensions and PHQ-9 (Patient Health Questionnaire-9) depression sum scores, mapped onto the MNI-152 (Montreal Neurological Institute) brain template. Higher z-scores indicate stronger relative associations across brain regions.

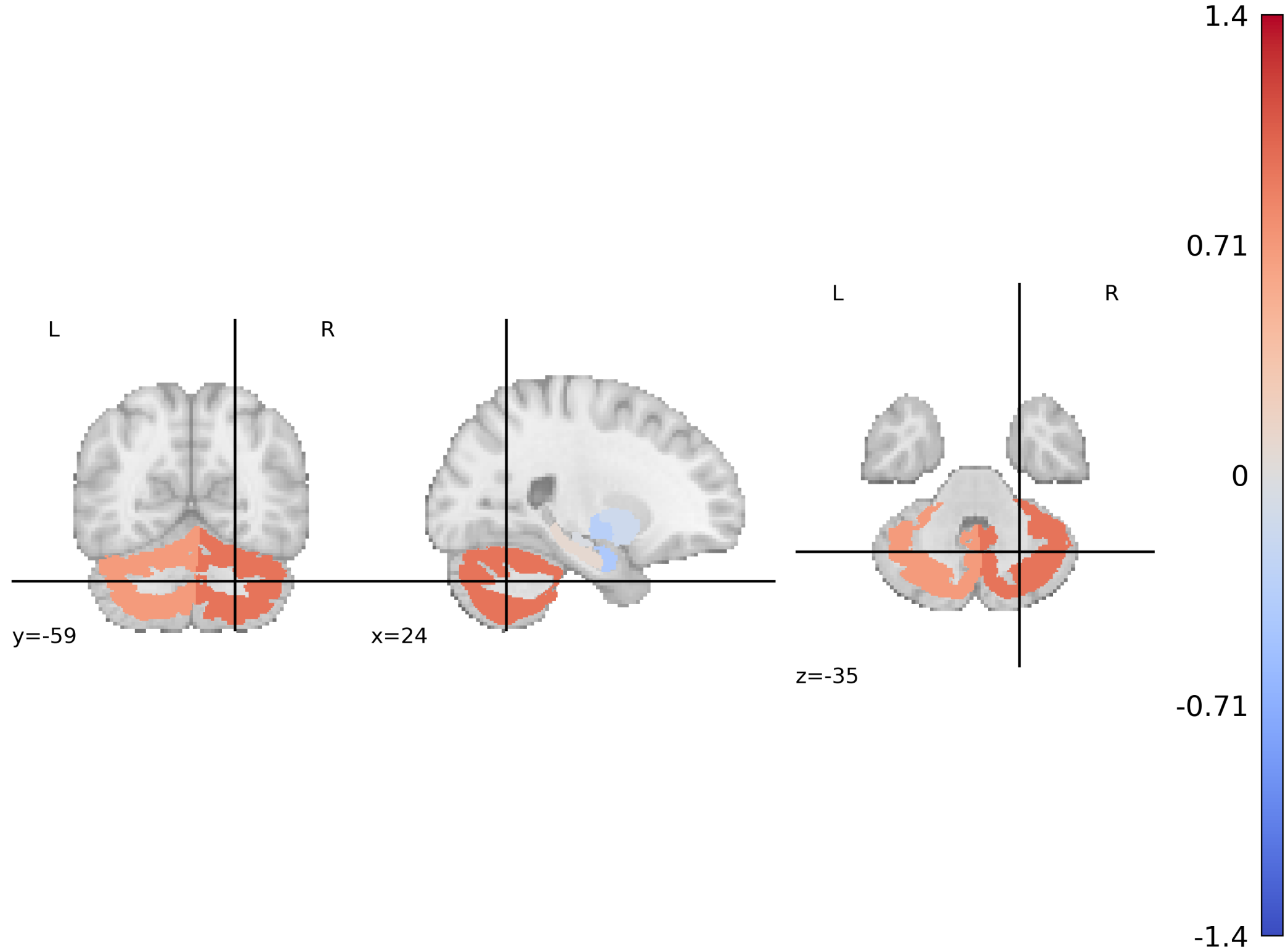

Figure 9: Z-scored heuristic associations between latent embedding dimensions and GAD-7 (Generalized Anxiety Disorder-7) anxiety sum scores, mapped onto the MNI-152 (Montreal Neurological Institute) brain template. Higher z-scores indicate stronger relative associations across brain regions.

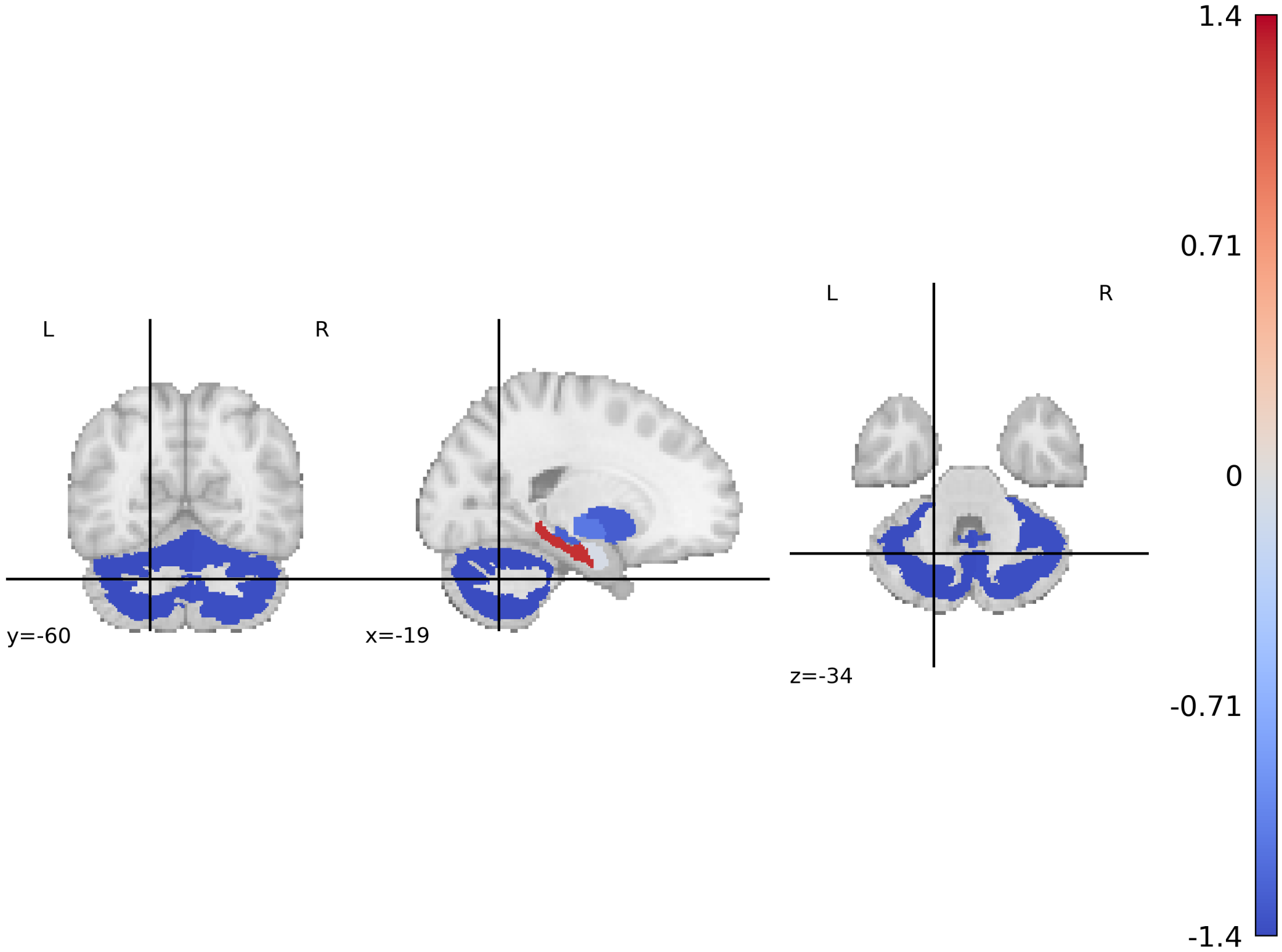

Figure 10: Z-scored heuristic associations between latent embedding dimensions and AUDIT-C (Alcohol Use Disorders Identification Test–Consumption) alcohol use sum scores, mapped onto the MNI-152 (Montreal Neurological Institute) brain template. Higher z-scores indicate stronger relative associations across brain regions.

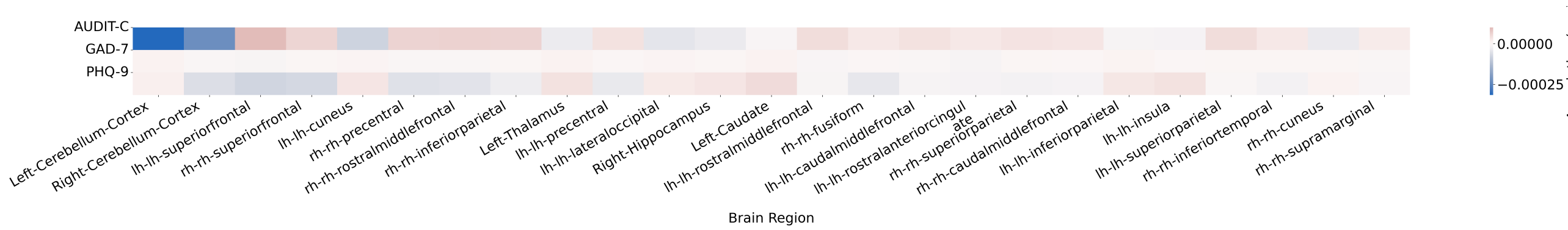

Figure 11: Top 25 brain regions ranked by absolute effect size of heuristic associations between latent embedding dimensions and symptom severity scores. Rows correspond to AUDIT-C (Alcohol Use Disorders Identification Test–Consumption), GAD-7 (Generalized Anxiety Disorder-7), and PHQ-9 (Patient Health Questionnaire-9). Columns indicate FreeSurfer-derived brain regions. Colors denote the relative strength and direction of association (blue = negative, red = positive), with the scale representing association strength.

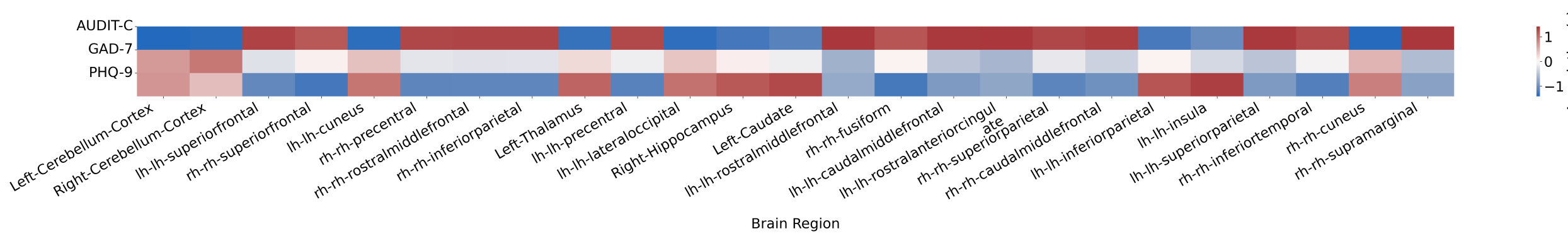

Figure 12: Top 25 brain regions ranked by absolute effect size of z-scored heuristic associations between latent embedding dimensions and symptom severity scores. Rows correspond to AUDIT-C (Alcohol Use Disorders Identification Test–Consumption), GAD-7 (Generalized Anxiety Disorder-7), and PHQ-9 (Patient Health Questionnaire-9). Columns indicate FreeSurfer-derived brain regions. Colors denote standardized association strength (z-scores), with blue indicating negative associations and red indicating positive associations. Z-scoring was applied across all regions to emphasize relative patterns.

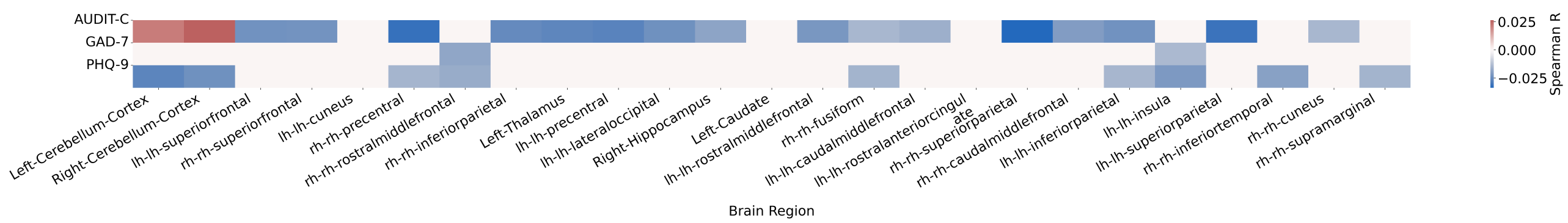

Figure 13: Top 25 brain regions ranked by absolute effect size of Spearman's ρ correlations between FreeSurfer-derived structural features and symptom severity scores. Rows correspond to AUDIT-C (Alcohol Use Disorders Identification Test–Consumption), GAD-7 (Generalized Anxiety Disorder-7), and PHQ-9 (Patient Health Questionnaire-9). Columns indicate individual FreeSurfer brain regions. Colors represent the strength and direction of correlations (blue = negative, red = positive), with intensity reflecting absolute correlation magnitude.

Table 14: ElasticNet-derived explained variance for significant embedding dimensions.

Score	Dimension	R2
AUDIT-C sum score	224	0.110
AUDIT-C sum score	162	0.395
AUDIT-C sum score	204	0.227
AUDIT-C sum score	155	0.280
AUDIT-C sum score	86	0.224
AUDIT-C sum score	73	0.131
AUDIT-C sum score	62	0.097
AUDIT-C sum score	87	0.229
AUDIT-C sum score	122	0.358
AUDIT-C sum score	216	0.194
AUDIT-C sum score	182	0.172
AUDIT-C sum score	102	0.328
AUDIT-C sum score	112	0.229
AUDIT-C sum score	223	0.200
AUDIT-C sum score	31	0.182
AUDIT-C sum score	177	0.290
AUDIT-C sum score	61	0.098
AUDIT-C sum score	173	0.108
GAD-7 sum score	100	0.098
GAD-7 sum score	241	0.378
GAD-7 sum score	217	0.215
GAD-7 sum score	58	0.281
PHQ-9 sum score	76	0.104
PHQ-9 sum score	217	0.215
PHQ-9 sum score	246	0.204
PHQ-9 sum score	100	0.098
PHQ-9 sum score	205	0.401
PHQ-9 sum score	132	0.267
PHQ-9 sum score	225	0.105
PHQ-9 sum score	231	0.336
PHQ-9 sum score	6	0.220
PHQ-9 sum score	233	0.262
PHQ-9 sum score	45	0.214
PHQ-9 sum score	91	0.223
PHQ-9 sum score	227	0.383
PHQ-9 sum score	216	0.194

3.7 Classification Baseline

Group	Confounders only		Freesurfer only	
	**BACC**	**AUC**	**BACC**	**AUC**
PHQ-9 (1)	58 ± 1	61 ± 1	58 ± 1	61 ± 1
PHQ-9 (2)	61 ± 2	65 ± 2	61 ± 3	65 ± 2
PHQ-9 (3)	57 ± 9	59 ± 15	65 ± 3	70 ± 3
PHQ-9 (4)	57 ± 16	59 ± 29	69 ± 7	76 ± 7
MDD ICD-10	57 ± 3	61 ± 3	58 ± 3	61 ± 3
MDD (diag.)	58 ± 1	61 ± 1	58 ± 1	61 ± 2
GAD-7 (2)	58 ± 2	61 ± 2	58 ± 2	61 ± 2
GAD-7 (3)	57 ± 5	61 ± 7	57 ± 4	61 ± 4
GAD-7 (4)	62 ± 7	66 ± 13	63 ± 4	69 ± 5
ANX ICD-10	56 ± 4	60 ± 4	57 ± 3	60 ± 3
ANX (diag.)	56 ± 2	58 ± 2	56 ± 2	58 ± 2
AUDIT-C (8)	60 ± 3	65 ± 3	60 ± 3	63 ± 3
AUDIT-C (9)	63 ± 3	68 ± 4	62 ± 4	66 ± 3
AUDIT-C (≥ 10)	63 ± 3	68 ± 3	64 ± 3	69 ± 3
AUD ICD-10	58 ± 8	57 ± 11	58 ± 5	64 ± 6

Table 15: Balanced accuracy (BACC; computed at a decision threshold of 0.5) and area under the receiver operating characteristic curve (AUC) for classification across all diagnostic outcomes in the UKB test set. Models included: *Confounders only* (sex, age, age-squared, and age–sex interaction), *FreeSurfer only* (Freesurfer-derived brain features with covariates). Values represent mean ± standard deviation across 10-fold stratified cross-validation.

3.8 Classification Performance Details

Table 16: Balanced accuracy (BACC; computed at a decision threshold of 0.5) distributions across 10-fold stratified cross-validation for all diagnostic outcomes. Columns show the minimum, maximum, mean, median, and standard deviation of BACC values across folds. Model configurations include: *Confounders only* (sex, age, age-squared, age–sex interaction), *Deviation 256d + covariates* (256-dimensional brain deviations with covariates).

Target	Config	Min	Max	Mean	Median	Std
MDD ICD-10	Confounders only	0.53	0.65	0.57	0.57	0.03
MDD ICD-10	Deviation 256d + covariates	0.58	0.67	0.62	0.62	0.03
PHQ-9 (1)	Confounders only	0.56	0.60	0.58	0.58	0.01
PHQ-9 (1)	Deviation 256d + covariates	0.56	0.61	0.59	0.59	0.02
PHQ-9 (2)	Confounders only	0.58	0.64	0.61	0.61	0.02
PHQ-9 (2)	Deviation 256d + covariates	0.60	0.67	0.63	0.62	0.02
PHQ-9 (3)	Confounders only	0.47	0.73	0.62	0.62	0.07
PHQ-9 (3)	Deviation 256d + covariates	0.51	0.68	0.61	0.61	0.06

Continued on next page

Target	Config	Min	Max	Mean	Median	Std
PHQ (4)	Confounders only	0.31	0.77	0.59	0.66	0.18
PHQ (4)	Deviation 256d + covariates	0.56	0.81	0.70	0.71	0.07
GAD (2)	Confounders only	0.55	0.60	0.58	0.59	0.02
GAD (2)	Deviation 256d + covariates	0.55	0.61	0.58	0.59	0.02
GAD (3)	Confounders only	0.53	0.62	0.58	0.57	0.03
GAD (3)	Deviation 256d + covariates	0.58	0.65	0.61	0.60	0.02
GAD (4)	Confounders only	0.53	0.70	0.65	0.67	0.05
GAD (4)	Deviation 256d + covariates	0.53	0.71	0.62	0.63	0.05
ANX ICD-10	Confounders only	0.50	0.60	0.56	0.56	0.03
ANX ICD-10	Deviation 256d + covariates	0.55	0.62	0.58	0.58	0.02
AUD ICD-10	Confounders only	0.49	0.65	0.57	0.56	0.06
AUD ICD-10	Deviation 256d + covariates	0.55	0.68	0.62	0.62	0.04
AUDIT-C (8)	Confounders only	0.56	0.64	0.60	0.59	0.03
AUDIT-C (8)	Deviation 256d + covariates	0.53	0.64	0.60	0.61	0.03
AUDIT-C (9)	Confounders only	0.58	0.68	0.63	0.64	0.04
AUDIT-C (9)	Deviation 256d + covariates	0.57	0.69	0.64	0.64	0.04
AUDIT-C (10)	Confounders only	0.57	0.68	0.63	0.63	0.03
AUDIT-C (10)	Deviation 256d + covariates	0.60	0.69	0.66	0.66	0.03
MDD (diag.)	Confounders only	0.56	0.60	0.58	0.58	0.01
MDD (diag.)	Deviation 256d + covariates	0.56	0.61	0.59	0.59	0.01
ANX (diag.)	Confounders only	0.53	0.59	0.56	0.56	0.02
ANX (diag.)	Deviation 256d + covariates	0.54	0.59	0.57	0.57	0.02

Table 17: Area under receiver operating characteristic curve (AUC) distributions across 10-fold stratified cross-validation for all diagnostic outcomes. Columns show the minimum, maximum, mean, median, and standard deviation of balanced accuracy (BACC; computed at a decision threshold of 0.5) values across folds. Model configurations include: *Confounders only* (sex, age, age-squared, age–sex interaction), *PRS + covariates* (polygenic risk scores with covariates), *Deviation 256d + covariates* (256-dimensional brain deviations with covariates), and *PRS + Deviation 256d + covariates* (combined model).

Target	Config	Min	Max	Mean	Median	Std
MDD ICD-10	Confounders only	0.53	0.68	0.61	0.61	0.04
MDD ICD-10	Deviation 256d + covariates	0.62	0.72	0.66	0.66	0.03
PHQ (1)	Confounders only	0.58	0.62	0.61	0.61	0.01
PHQ (1)	Deviation 256d + covariates	0.59	0.64	0.61	0.61	0.01
PHQ (2)	Confounders only	0.62	0.71	0.65	0.65	0.02
PHQ (2)	Deviation 256d + covariates	0.63	0.70	0.67	0.67	0.02
PHQ (3)	Confounders only	0.48	0.75	0.68	0.69	0.08
PHQ (3)	Deviation 256d + covariates	0.55	0.74	0.66	0.68	0.07
PHQ (4)	Confounders only	0.13	0.89	0.59	0.71	0.30

Continued on next page

Target	Config	Min	Max	Mean	Median	Std
PHQ (4)	Deviation 256d + covariates	0.64	0.86	0.75	0.74	0.08
GAD (2)	Confounders only	0.58	0.64	0.61	0.62	0.02
GAD (2)	Deviation 256d + covariates	0.59	0.64	0.61	0.62	0.02
GAD (3)	Confounders only	0.57	0.67	0.63	0.63	0.03
GAD (3)	Deviation 256d + covariates	0.58	0.69	0.63	0.63	0.03
GAD (4)	Confounders only	0.55	0.77	0.70	0.73	0.07
GAD (4)	Deviation 256d + covariates	0.54	0.73	0.65	0.65	0.06
ANX ICD-10	Confounders only	0.52	0.65	0.59	0.59	0.05
ANX ICD-10	Deviation 256d + covariates	0.58	0.68	0.63	0.63	0.04
AUD ICD-10	Confounders only	0.46	0.68	0.59	0.62	0.08
AUD ICD-10	Deviation 256d + covariates	0.52	0.70	0.65	0.66	0.05
AUDIT-C (8)	Confounders only	0.58	0.70	0.65	0.64	0.04
AUDIT-C (8)	Deviation 256d + covariates	0.57	0.70	0.65	0.64	0.04
AUDIT-C (9)	Confounders only	0.61	0.75	0.68	0.69	0.04
AUDIT-C (9)	Deviation 256d + covariates	0.63	0.76	0.69	0.69	0.04
AUDIT-C (10)	Confounders only	0.62	0.71	0.68	0.69	0.03
AUDIT-C (10)	Deviation 256d + covariates	0.65	0.75	0.72	0.72	0.03
MDD (diag.)	Confounders only	0.58	0.63	0.61	0.61	0.02
MDD (diag.)	Deviation 256d + covariates	0.60	0.64	0.62	0.62	0.01
ANX (diag.)	Confounders only	0.55	0.62	0.58	0.58	0.02
ANX (diag.)	Deviation 256d + covariates	0.55	0.62	0.60	0.60	0.02

Bibliography

[1] Kaiming He, Haoqi Fan, Yuxin Wu, Saining Xie, and Ross Girshick. Momentum contrast for unsupervised visual representation learning. In *Proceedings of the IEEE/CVF conference on computer vision and pattern recognition*, pages 9729–9738, 2020. doi: 10.1109/CVPR42600.2020.00975.

[2] Xinlei Chen, Saining Xie, and Kaiming He. An empirical study of training self-supervised vision transformers. In *2021 IEEE/CVF International Conference on Computer Vision (ICCV)*, pages 9620–9629, 2021. doi: 10.1109/ICCV48922.2021.00950.

[3] Junnan Li, Ramprasaath R. Selvaraju, Akhilesh D. Gotmare, Shafiq Joty, Caiming Xiong, and Steven C.H. Hoi. Align before fuse: vision and language representation learning with momentum distillation. In *Proceedings of the 35th International Conference on Neural Information Processing Systems*, NIPS '21, Red Hook, NY, USA, 2021. Curran Associates Inc. ISBN 9781713845393.

[4] Jielin Qiu, Jiacheng Zhu, Shiqi Liu, William Han, Jingqi Zhang, Chaojing Duan, Michael A. Rosenberg, Emerson Liu, Douglas Weber, and Ding Zhao. Automated cardiovascular record retrieval by multimodal learning between electrocardiogram and clinical report. In Stefan Hegselmann, Antonio Parziale, Divya Shanmugam, Shengpu Tang, Mercy Nyamewaa Asiedu, Serina Chang, Tom Hartvigsen, and Harvineet Singh, editors, *Proceedings of the 3rd Machine Learning for Health Symposium*, volume 225 of *Proceedings of Machine Learning Research*, pages 480–497. PMLR, 10 Dec 2023. URL `https://proceedings.mlr.press/v225/qiu23a.html`.

[5] Fernando Pérez-García, Rachel Sparks, and Sébastien Ourselin. TorchIO: A python library for efficient loading, preprocessing, augmentation and patch-based sampling of medical images in deep learning. *Computer Methods and Programs in Biomedicine*, 208:106236, 2021. doi: 10.1016/j.cmpb.2021.106236.

[6] Adam Paszke, Sam Gross, Soumith Chintala, Gregory Chanan, Edward Yang, Zachary DeVito, Zeming Lin, Alban Desmaison, Luca Antiga, and Adam Lerer. Automatic differentiation in PyTorch. 2017.

[7] Bruce Fischl. Freesurfer. *NeuroImage*, 62(2):774–781, 2012. ISSN 1053-8119. doi: 10.1016/j.neuroimage.2012.01.021. 20 YEARS OF fMRI.

[8] B.W. Silverman. *Density Estimation for Statistics and Data Analysis*. 02 2018. ISBN 9781315140919. doi: 10.1201/9781315140919.

[9] E. WEISZFELD. Sur le point pour lequel la somme des distances de n points donnés est minimum. *Tohoku Mathematical Journal, First Series*, 43:355–386, 1937.

[10] John D. Storey. A direct approach to false discovery rates. *Journal of the Royal Statistical Society Series B: Statistical Methodology*, 64(3):479–498, 2002. doi: 10.1111/1467-9868.00346.
